# Screening Key Genes Related to Nitrogen Use Efficiency in Cucumber Through Weighted Gene Co-Expression Network Analysis

**DOI:** 10.3390/genes15121505

**Published:** 2024-11-24

**Authors:** Linhao Ma, Aimin Wei, Ce Liu, Nan Liu, Yike Han, Zhengwu Chen, Ningning Wang, Shengli Du

**Affiliations:** 1 College of Life Science, Nankai University, Tianjin 300071, Chinawangnn@nankai.edu.cn (N.W.); 2 Cucumber Research Institute, Tianjin Academy of Agricultural Sciences, Tianjin 300192, China; 3 State Key Laboratory of Vegetable Biobreeding, Tianjin Academy of Agricultural Sciences, Tianjin 300381, China; 4 College of Agricultural Science, Nankai University, Tianjin 300071, China

**Keywords:** cucumber, NUE, transcriptomic sequencing, WGCNA, key genes

## Abstract

Cucumber (*Cucumis sativus* L.) is a crucial vegetable crop, requiring significant nitrogen fertilizer inputs. However, excessive nitrogen application not only impairs growth but also poses severe environmental risks. Thus, enhancing nitrogen use efficiency (NUE) in cucumber is imperative. For the identification of genes associated with NUE in cucumber, roots of high NUE and low NUE lines were analyzed under high nitrogen conditions. Using transcriptome sequencing through WGCNA, a total of 15,180 genes were categorized into 35 co-expression modules, with 5 modules being highly correlated with NUE. Based on differential expression within the five modules and the results of GO and KEGG enrichment analyses, 25 genes were identified as potentially related to NUE. Among these, *CsaV4_1G002492* (GLR22), *CsaV4_2G003460* (GLR35), *CsaV4_3G000307* (NRT1.1), and *CsaV4_7G001709* (UPS2) were homologous to genes in *Arabidopsis* known to directly participate in NUE related process. These four genes were chosen as key genes for further analysis. qRT-PCR analysis revealed that *CsaV4_3G000307* and *CsaV4_7G001709* were more active during the early stages of the high nitrogen treatment in the high NUE line. Conversely, *CsaV4_1G002492* and *CsaV4_2G003460* were more active in the low NUE line. Using transcriptomic analysis, a frameshift INDEL mutation was observed in *CsaV4_3G000307* in the low NUE line, which impacted the compactness of the protein structure, potentially altering its function. Analysis of protein interactions of these four key genes predicted some potential interaction networks. This research offers critical insights into the genetic factors influencing NUE in cucumber, presenting potential targets for genetic modification or breeding programs.

## 1. Introduction

Cucumber is one of the top ten vegetables globally, widely cultivated across various regions, and plays a significant role as an economic crop with substantial impacts on global agriculture [1]. According to the Food and Agriculture Organization (FAO) (http://agris.fao.org/), the total production area of cucumbers worldwide was 2.174 million hectares with a total yield of 94.718 million tons in 2022. Cucumber is a crop with high nutrient demands, particularly with a significant requirement for nitrogen fertilizers [2]. The application of nitrogen fertilizers significantly increases cucumber yield and is crucial for achieving high productivity [3,4]. However, excessive application of nitrogen fertilizer inhibits growth of cucumbers and increase economic costs [5,6,7]. With increasing nitrogen fertilizer application, the NUE of cucumbers tends to decline [8,9]. In Chinese agricultural production, the average utilization rate of nitrogen fertilizers is only 30–35%, meaning that approximately 65–70% of the nitrogen is not effectively used by the crops [10]. This results not only in substantial economic losses but also in severe environmental issues, such as water eutrophication, groundwater pollution, greenhouse gas emissions, and soil acidification [11,12,13,14]. Consequently, optimizing nitrogen fertilizer application and enhancing NUE are imperative for sustainable agricultural development.

However, the processes of nitrogen response and efficiency are governed by intricate networks involving metabolic, developmental, and environmental signals. Considerations of nitrogen absorption, transport, assimilation, and extensive signaling networks are necessary for enhancing NUE. Robust modern strategies are employed for identifying key genes. Chu et al. used genomic positional mapping and pinpointed a gene critical for high nitrate utilization, *NRT1.1b*. A SNP mutation in this gene has been demonstrated to influence nitrogen use efficiency in rice [15]. Zhou et al., through comparative transcriptomic and metabolomic analysis of maize and rice leaf tissues, pinpointed the gene *OsDREB1C*, which was involved in augmenting photosynthesis and NUE [16]. Gao et al. demonstrated that analysis of the expression profile of *CsNRT2.1* showed that *CsNRT2.1* is a high affinity nitrate transporter [17]. Ming et al. analyzed the expression patterns and subcellular localization of the GS1 protein under different nitrogen conditions, confirming its role in enhancing low nitrogen tolerance in cucumber [18]. Transcriptomic analysis through WGCNA is considered a powerful means of mining key genes [19]. WGCNA categorizes a multitude of genes into different modules based on gene expression changes, reducing analytical complexity and associating co-expression patterns within modules with phenotypic differences, thus identifying gene modules and key genes that play crucial roles during phenotypic variations [20,21,22,23]. This method has been extensively applied to unravel molecular mechanisms in various plants, including cucumbers [24]. Examples include identifying key genes responsible for resistance against *Podosphaera xanthii* in cucumber cultivars [25], pinpointing genes crucial for phenylpropanoid biosynthesis during cucumber storage [26], determining central genes for salt response [27], and identifying genes significantly associated with flavonoid biosynthesis in cucumbers [28]. Moreover, SNPs and INDELs are genetic variations that can significantly influence phenotypic traits [15,29,30]. The accurate identification of these mutations is crucial for genetic research and breeding programs. The presence of an SNP or INDEL may lead to phenotypic changes, playing a significant role in genetic breeding [31]. For instance, SNPs and INDELs have been used to assist in selecting genes resistant to powdery mildew [32], genes resistant to stem blight [33], genes associated with germination [34], and key genes for parthenocarpy in cucumbers [35].

Previously, we have established laboratory techniques for identifying phenotypes under varying nitrogen concentrations during the seedling stage, and yielded materials with extreme genotypes. Five candidate genes associated with cucumber tolerance to low nitrogen were identified through genome-wide association studies [36]. In this study, two extreme phenotypic cucumber lines under high nitrogen conditions were used. Transcriptomic sequencing of root tissues at seven time points after high nitrogen treatment was performed. Using the WGCNA method, a co-expression network of genes related to nitrogen use efficiency under high nitrogen conditions was constructed. Specific modules responding to NUE in cucumber were selected, and their functions were annotated and analyzed to mine related key genes and interaction networks. This analysis offers new insights into the regulation mechanisms of NUE in cucumber.

## 2. Materials and Methods

### 2.1. Plant Materials and High Nitrogen Treatment

Two extreme phenotypic cucumber lines, L2 with high NUE and L8 with low NUE, were used as plant material, which were obtained through previous screening of 112 cucumber lines of different cucumber types at Cucumber Research Institute of Tianjin Academy of Agricultural Sciences, China. L2 and L8 both belong to the South China cucumber type. Seeds were initially soaked in warm water at 55 °C for 15 min, followed by immersion in room temperature water for 3.5 h. Subsequently, seeds were placed in a germination culture box maintained at 28 °C for 2–3 days to allow sprout development. The sprouts were affixed to sponge plugs and then were transferred to a plastic box and cultivated in a half-strength Hoagland nutrient solution (nitrate concentration of 7 mmol/L) at a pH of 6.2. The nutrient solution was refreshed every three days and aeration was conducted twice a day. Once seedlings reached the one true leaf stage, they were transferred to 700 mL black hydroponic plastic buckets containing 500 mL of half-strength Hoagland nutrient solution. When seedlings reached the two true leaf stage, they were subjected to a higher nitrate treatment with a nitrogen concentration of 20 mmol/L. The Hoagland nutrient solution recipe is available in the Supplementary Table S1. Root sampling was conducted at 0 h, 2 h, 4 h, 8 h, 12 h, 24 h, and 30 h of cultivation, with at least three biological replicates per treatment.

### 2.2. RNA Extraction and Transcriptome Sequencing

Total RNA was extracted from root tissues using the standard extraction method provided by Novogene Co. Ltd. The quality and integrity of RNA were assessed with an Agilent 2100 bioanalyzer. Libraries for transcriptomic sequencing were prepared using the standard NEB library construction kit [37] and were sequenced on the Illumina platform to generate paired-end reads. Raw sequence reads were processed and aligned to the cucumber reference V4 genome (http://www.cucumberdb.com/#/home, accessed on 25 September 2024) utilizing HISAT2 [38].

### 2.3. Weighted Gene Co-Expression Network Analysis

Transcriptomic data from 14 samples were analyzed using the Novamagic cloud platform with a soft threshold set at 10. Genes were hierarchically clustered based on their Topological Overlap Matrix (TOM) dissimilarity [19], and modules were identified using dynamic tree cutting techniques. Each module contained a minimum of 30 genes. Modules that exhibited highly similar expression profiles (dissimilarity less than 0.25) were merged to minimize redundancy and enhance the robustness of the findings.

### 2.4. Differential Expression Genes Analysis

Differentially expressed genes (DEGs) between the high NUE and low NUE lines at each time point were identified using the edgeR [39]. The *p*-values were adjusted using the Benjamini and Hochberg method. Corrected *p*-value of 0.05 and absolute log_2_(foldchange) of 0.8 were set modules as the thresholds for significantly differential expression. A DEGs Venn diagram was created using an R 4.4.1 package.

### 2.5. Identification and Enrichment Analysis of the Core Modules

Identification and enrichment analysis of the core modules were conducted by calculating the Pearson correlation coefficients and the significance of the p-values for the module eigengenes in relation to L2 and L8. Modules were selected as core if they exhibited a correlation coefficient (r) greater than 0.6 and a *p*-value less than 0.05. Differentially expressed genes (DEGs) within these modules were then analyzed using Gene Ontology (GO, http://www.geneontology.org/, accessed on 11 October 2024) and the Kyoto Encyclopedia of Genes and Genomes (KEGG, http://www.genome.jp/kegg/, accessed on 11 October 2024) to elucidate the biological functions and pathways predominantly represented. Bar charts were utilized to display the top 30 significant GO enrichment analysis results. Bubble charts were employed to show the top 20 KEGG pathway analysis results.

### 2.6. Real-Time Quantitative PCR Analysis

Primers were designed using NCBI (https://www.ncbi.nlm.nih.gov/, accessed on 1 October 2024) and synthesized by Sangon Biotech Co., Ltd. (Shanghai, China). Detailed primer sequence information is available in Supplementary Table S2. RNA extraction was performed using the NG312 kit from Beijing LABLEAD Company, Beijing, China, and cDNA was synthesized with a First-strand cDNA Synthesis Mix kit from the same company. All procedures were carried out according to the instructions provided. The qRT-PCR was conducted using the 2x Realab Green PCR Fast mixture reagent from Beijing LABLEAD Company, Beijing, China in a 20 µL system as per the manufacturer’s instructions. The protocol included an initial denaturation at 95 °C for 30 s, followed by 40 cycles of denaturation at 95 °C for 10 s and annealing at 60 °C for 30 s. A melting curve analysis was performed from 60 to 90 °C in 10 s intervals with a ∆T of 0.5 °C. An actin gene served as the internal control for data standardization, with relative expression calculated using the 2^−∆∆Ct^ method. Variance analysis was conducted using R with a *t*-test to determine significance.

### 2.7. Transcriptomic SNPs and INDELs Analysis

SNPs and INDELs were analyzed using the Novamagic cloud platform. Sequence logos were generated to visualize these variations using the WebLogo tool, providing a graphical representation of sequence conservation and variation at identified loci [40,41].

### 2.8. Protein Structure and Interaction Prediction

Protein structure prediction was performed using AlphaFold3, with the default settings for speed and copies parameters [42]. The detailed protein sequence is in Supplementary File S1. STRING was used to predict protein interaction, employing default settings (https://cn.string-db.org/, accessed on 26 October 2024).

## 3. Results

### 3.1. Identification of Gene Co-Expression Modules Through WGCNA

**Figure 1:**
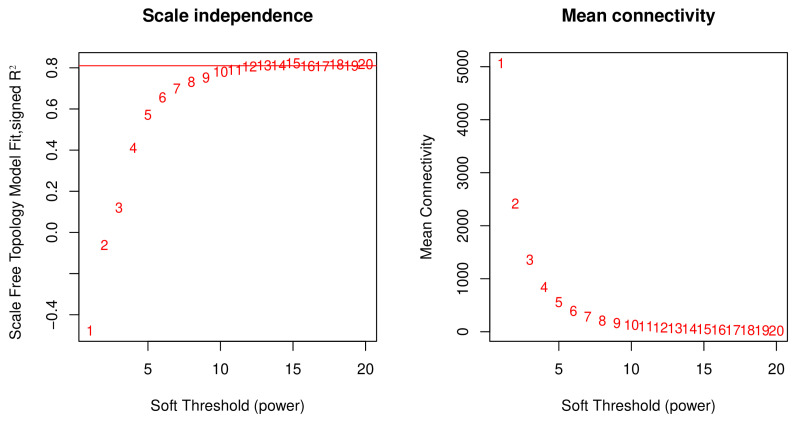
Soft-threshold power selection for gene co-expression network construction. Left panel: red line represents scale-free topology fit index across varying soft-threshold powers. Right panel: mean connectivity changes with increasing soft-threshold power.

An optimal soft-threshold power was selected for the construction of the gene co-expression network by assessing the scale-free topology. Various soft-threshold powers, ranging from 1 to 20, were evaluated (Figure 1). A power of 10 was determined to be optimal, corresponding to a fit index that exceeded 0.8 and ensuring stabilization and the attainment of a scale-free topology within the network.

Initial clustering based on the TOM revealed an extensive array of color blocks, reflecting a complex pattern of gene co-expressions that complicated further analysis (Supplementary Figure S1). The clustering led to the identification of 35 gene co-expression modules, which varied significantly in size. The smallest module, labeled grey, contained 48 genes, while the largest, labeled turquoise, included over 4000 genes, demonstrating the diversity of gene co-expression across the sampled conditions.

### 3.2. Correlation Between the Modules and NUE

**Figure 2:**
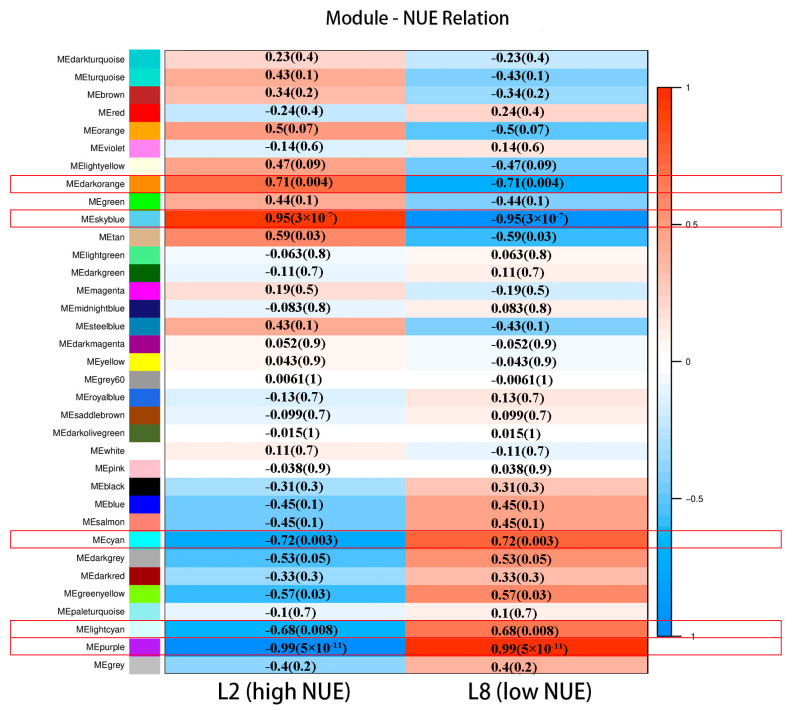
Correlation heatmap between gene co-expression modules and L2/L8 lines. Red indicates positive correlations and blue denotes negative correlations. Cells contain Pearson correlation coefficients and *p*-values in parentheses.

To elucidate the roles of the 35 identified modules in the two lines, associations were explored by calculating Pearson correlation coefficients between module eigengenes and the L2 (high NUE)/ L8 (low NUE) lines. As shown in Figure 2, a detailed heatmap illustrates the strength and significance of these correlations, highlighting each module’s potential contribution to NUE. Five out of the 35 modules identified were found to be significantly correlated with the extreme NUE lines. The purple (correlation coefficient 0.99), skyblue (correlation coefficient 0.95), cyan (correlation coefficient 0.72), darkorange (correlation coefficient 0.71), and lightcyan (correlation coefficient 0.68) modules, contain 241, 86, 200, 101, and 195 genes, respectively.

Modules such as darkorange and skyblue, which show positive correlations, were considered to be active in the high NUE line, whereas modules like purple, cyan, and lightcyan, showing negative correlations, were considered to be active in the low NUE line, all of which were targeted for further comprehensive functional studies.

### 3.3. Comprehensive Analysis of the Core Modules

#### 3.3.1. Analysis of the Purple Module

**Figure 3:**
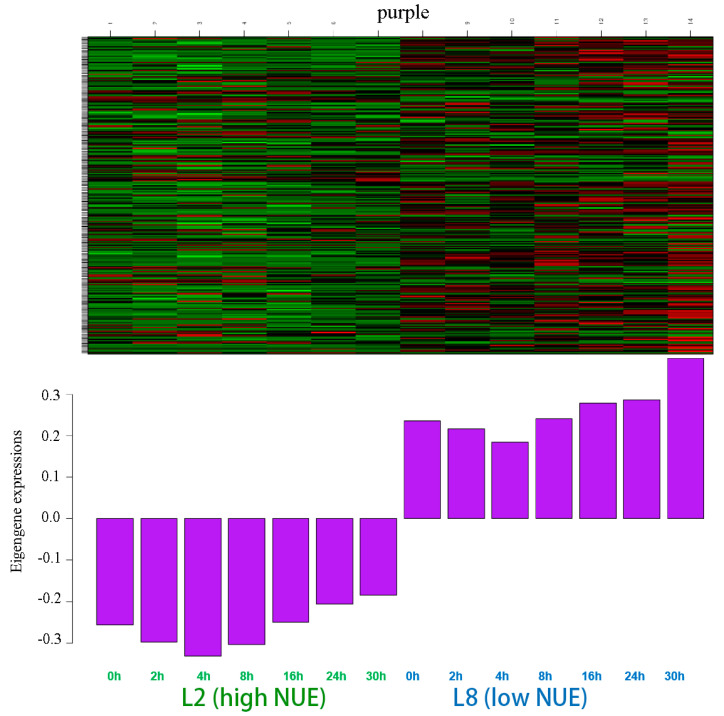
Heatmap of the gene expression of the eigengenes in the purple module. Color ranges from green (low) to red (high).

The purple module, encompassing 241 genes, displayed distinct expression patterns across the seven different time points in response to high nitrogen. Figure 3 illustrates that eigengene expressions differ significantly between the high NUE and low NUE cucumber lines. A peak in expression at 4 h in the high NUE line corresponded to the lowest expression in the low NUE line, highlighting significant differences in high nitrogen response.

The maximum number of DEGs within this module was observed at 4 h, totaling 145 genes (Supplementary Figure S2). The time points at 2 and 8 h each recorded 140 DEGs, while the lowest count of 129 DEGs occurred at 24 h. Seventy-four genes were differentially expressed at all time points, underlining their possible vital role in nitrogen metabolism.

GO (Supplementary Figure S3 and Table S3) and KEGG enrichment analyses (Supplementary Figure S4 and Table S4), elucidated the biological functions and pathways of these DEGs. The enrichment analysis highlighted several crucial biological processes and molecular functions related to NUE. Key NUE-related functions and pathways included glycine, serine, and threonine metabolism, aminoacyl-tRNA biosynthesis, and arginine biosynthesis and metabolism. Fifteen genes were identified, with nine exhibiting differential expression at four or more time points. These nine genes were selected as candidate genes for further analysis.

#### 3.3.2. Analysis of the Lightcyan Module

**Figure 4:**
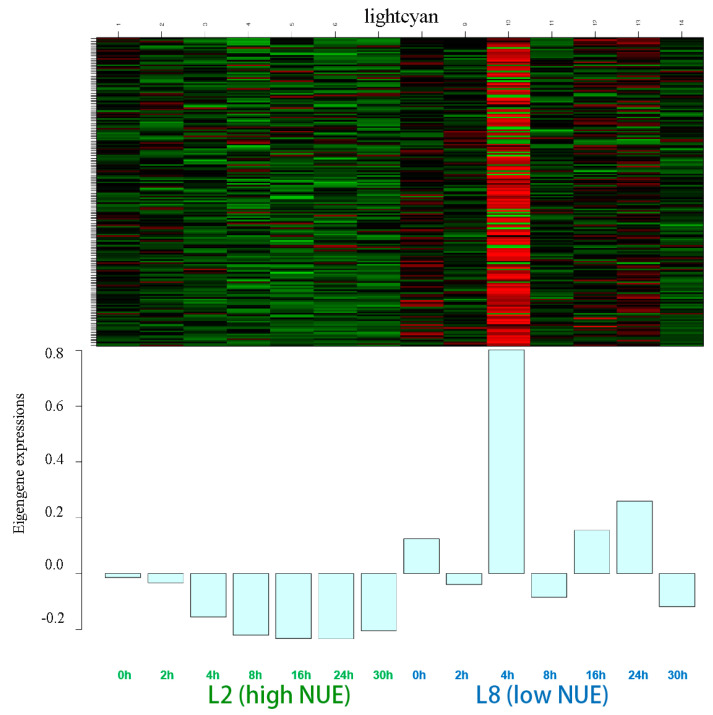
Gene expression of the eigengenes in the lightcyan module. Color ranges from green (low) to red (high).

The lightcyan module, encompassing 195 genes, exhibited diverse expression patterns across the seven time points (Figure 4). Significant differences in expression between the high NUE and low NUE lines were observed, which was particularly notable at 4 h where the low NUE line displayed remarkable peak eigengene expressions in contrast to the lower expressions in the high NUE line.

Within this module, the highest number of DEGs was observed at 4 h, totaling 147 genes (Supplementary Figure S5). This peak was followed by 98 DEGs at 24 h, with the lowest count of 29 DEGs at 2 h. While DEG counts at time points 0, 8, 12, and 30 h were recorded at 35, 63, 82, and 55, respectively. Notably, 74 genes were differentially expressed at four or more time points, with 7 genes differentially expressed across all time points.

GO enrichment identified 11 genes with NUE-related functions (Supplementary Figure S6 and Table S5) and KEGG pathways enrichment identified 10 genes (Supplementary Figure S7 and Table S6). After excluding duplicates, the enrichment analysis highlighted 19 genes with several crucial biological processes and molecular functions related to NUE, including arginine and proline metabolism, as well as glycine, serine, and threonine metabolism. Among these, 10 genes exhibited differential expression at four or more time points, emphasizing their potential roles in nitrogen metabolism. These 10 genes were selected as candidate genes for further analysis.

#### 3.3.3. Analysis of the Darkorange Module

**Figure 5:**
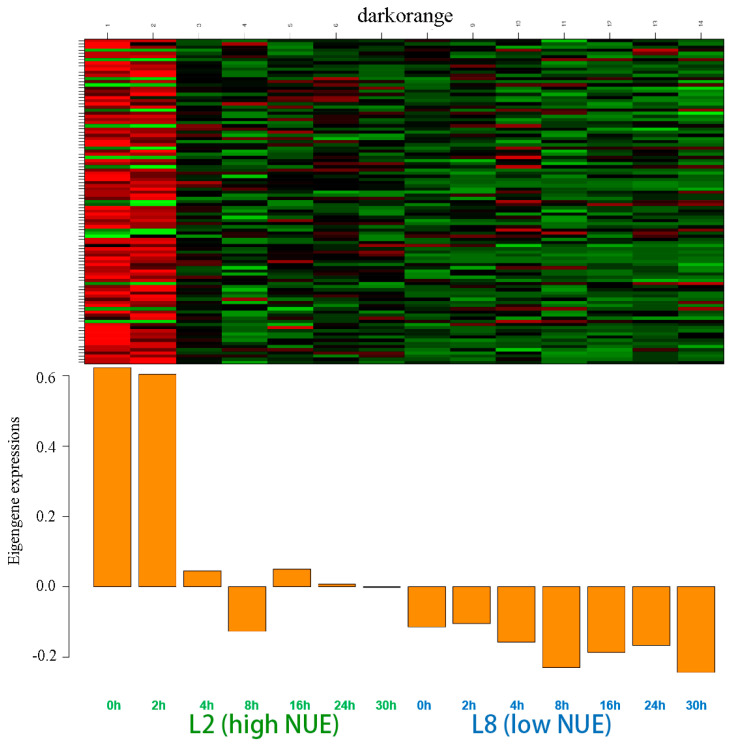
Gene expression of the eigengenes in the darkorange module. Color ranges from green (low) to red (high).

The darkorange module encompassed 101 genes. Notably higher eigengene expressions were observed at 0 and 2 h in the high NUE line, marking these periods as critical for gene activity within this module. In contrast, the low NUE line exhibited negative values at these times (Figure 5).

The number of DEGs peaked at 0 h with 64 genes, followed by 58 at 2 h (Supplementary Figure S8). The lowest differential expression was noted at 4 h with 23 genes, with consistent counts of 30 genes observed at both 8 and 12 h, and varying counts of 29 and 39 genes at 24 and 30 h, respectively. A total of 35 genes were identified as differentially expressed at four or more time points, where 12 exhibited differential expression across all seven time points.

GO enrichment identified 10 genes with NUE-related functions (Supplementary Figure S9 and Table S7) and KEGG pathways enrichment identified 5 genes (Supplementary Figure S10 and Table S8). These analyses highlighted crucial pathways such as nitrogen metabolism and arginine biosynthesis, which were essential for understanding nitrogen utilization in plants. After excluding duplicates, 14 genes critical for nitrogen utilization were identified, where 5 genes showed differential expression across four or more time points, underscoring their significant roles in nitrogen metabolism. These five genes were selected as candidate genes from this module for further analysis.

#### 3.3.4. The Analysis of the Cyan Module

**Figure 6:**
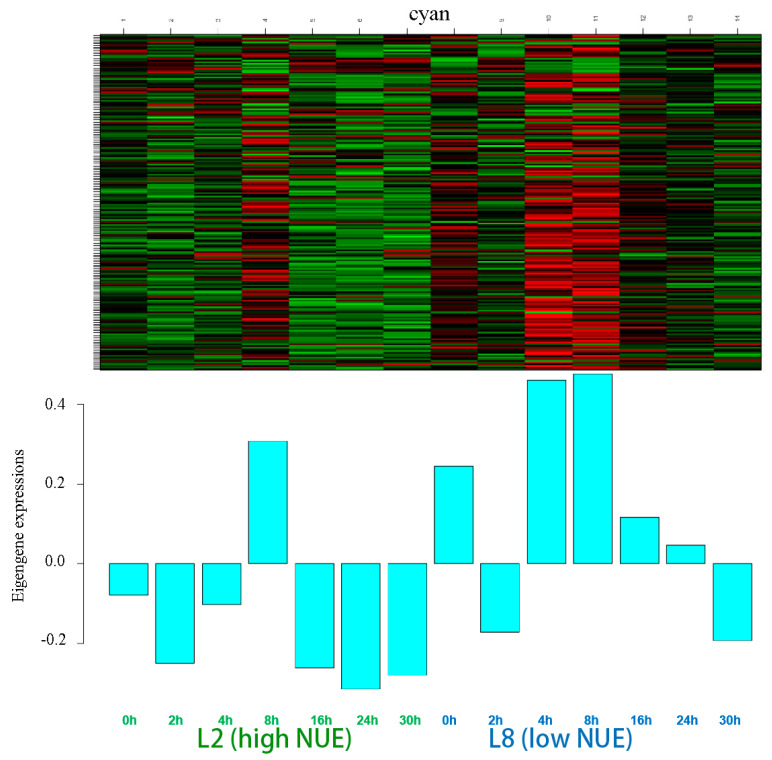
Gene expression of the eigengenes in the cyan module. Color ranges from green (low) to red (high).

The cyan module comprised 200 genes. Marked differences in eigengene expressions between the high NUE and low NUE lines were particularly prominent at 4 h, where the low NUE line showed a peak in positive values, which is in stark contrast to the negative values observed in the high NUE line (Figure 6).

The module exhibited a peak in differential expression at 4 h with 62 genes, and a secondary peak at 24 h with 47 genes (Supplementary Figure S11). The lowest differential expression was noted at 2 h with only 16 DEGs, and variable DEG counts were observed at other time points—29 at 0 h, 22 at 8 h, 35 at 12 h, and 36 at 30 h. Of these, 17 genes were identified as differentially expressed at four or more time points, with one gene expressed across all seven time points.

GO (Supplementary Figure S12 and Table S9) and KEGG pathways (Supplementary Figure S13 and Table S10) revealed the involvement of these genes in crucial metabolic pathways, including amino acid biosynthesis and arginine and proline metabolism. These pathways played vital roles in the plant metabolic framework, indirectly influencing nitrogen assimilation and utilization. However, there were no genes differentially expressed across four or more time points and therefore no genes were selected as candidate genes.

#### 3.3.5. The Analysis of the Skyblue Module

**Figure 7:**
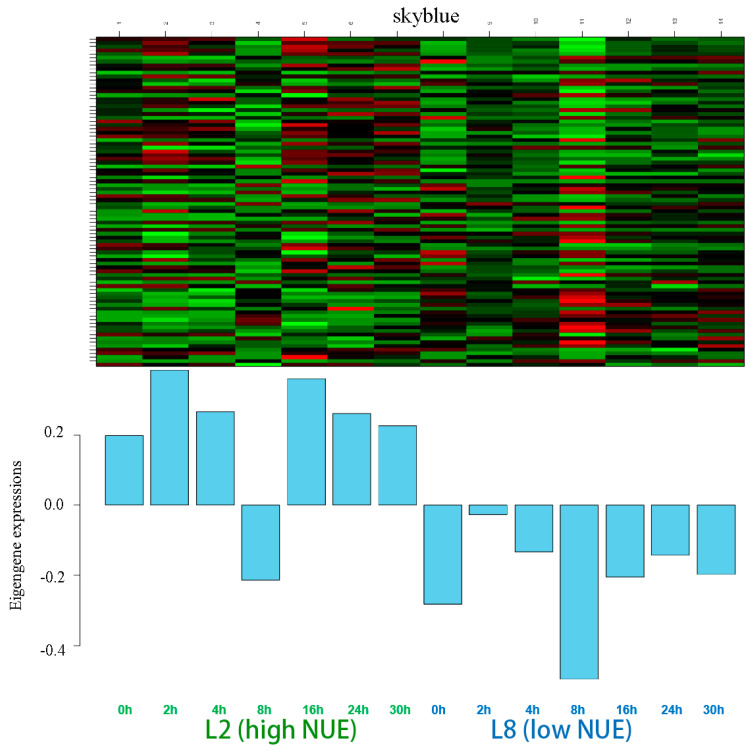
Gene expression of the eigengenes in the skyblue module. Color ranges from green (low) to red (high).

The skyblue module comprised 86 genes. The expression heatmap revealed no distinct time-specific variations within this module (Figure 7). A peak in differential expression was observed at 12 h involving 31 genes, closely followed by 29 genes at 4 h (Supplementary Figure S14). The lowest number of differentially expressed genes, totaling 20, was recorded at 8 h. Counts of differentially expressed genes at 0 and 30 h included 28 genes each, with 25 and 26 genes at 2 and 24 h, respectively. Importantly, 17 genes in this module were identified as differentially expressed across four or more time points, while 6 genes differentially expressed at all time points.

GO enrichment identified two genes with NUE-related functions (Supplementary Figure S15 and Table S11) and KEGG pathways enrichment identified one gene (Supplementary Figure S16 and Table S12). Crucial functions and pathways included nitrogen compound transport and arginine and proline metabolism. Among these genes, only one gene showed differential expression across multiple time points. This one gene was selected as a candidate gene from this module for further analysis.

### 3.4. Information and Functional Annotation of 25 Selected Genes

Table 1 provides detailed information about the candidate genes from the five modules, including gene descriptions, species affiliations, and their respective expression modules. These genes have been studied across multiple species, such as in *Arabidopsis thaliana*, *Oryza sativa*, *Cucumis melo*, *Cucumis sativus*, and so on. We further screened 25 genes by reviewing related studies to determine whether the genes or their gene family members had been previously studied for functions related to NUE. The results identified genes directly related to NUE such as *CsaV4_1G002492* (GLR22) and *CsaV4_2G003460* (GLR35), which are glutamate receptors potentially involved in nitrogen signal transduction; *CsaV4_3G000307* (NRT1.1/NPF6.3), which is involved in nitrogen absorption and transport; and *CsaV4_7G001709* (UPS2), which is involved in nitrogen recycling. These four genes were selected as key genes for further analysis.

### 3.5. Expression Profile Analysis of the Key Genes

**Figure 8:**
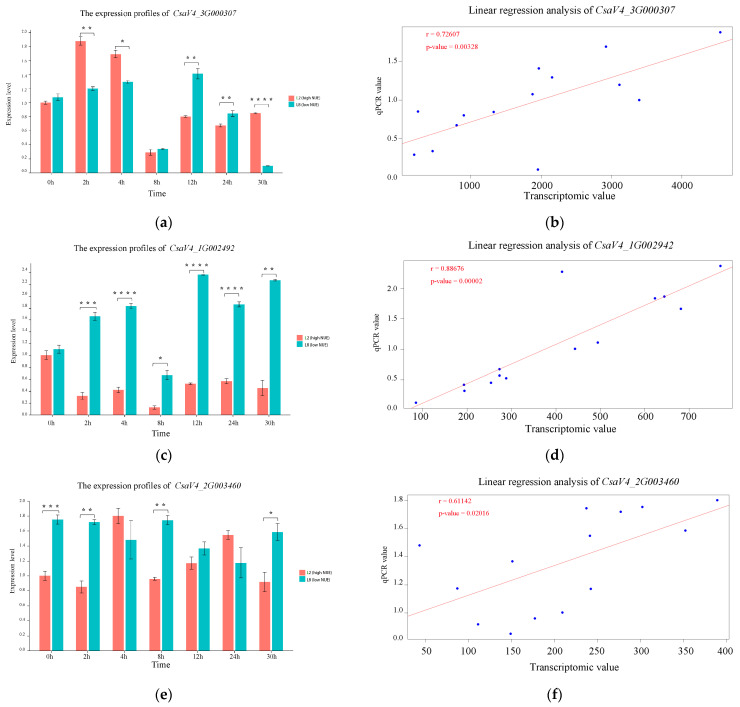

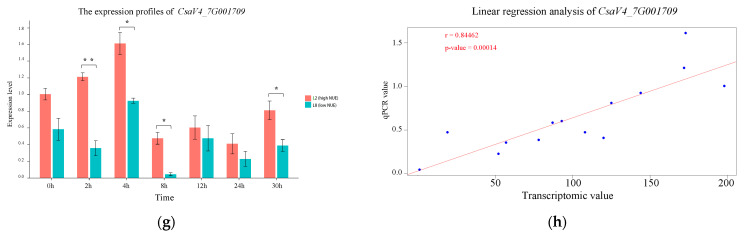
Expression profiles and the linear regression analysis of the four key genes. The expression profiles of (**a**) *CsaV4_3G000307*; (**c**) *CsaV4_1G002492*; (**e**) *CsaV4_2G003460*; and (**g**) *CsaV4_7G001709*. The linear regression analysis of (**b**) *CsaV4_3G000307*; (**d**) *CsaV4_1G002492*; (**f**) *CsaV4_2G003460*; and (**h**) *CsaV4_7G001709*. ‘r’ indicates the Pearson correlation coefficient and ‘*p*’ denotes significance. One ⭐ indicates a significance difference with *p* < 0.05, two ⭐ indicate *p* < 0.01, three ⭐ indicate *p* < 0.001, four ⭐ indicate *p* < 0.0001.

As shown in Figure 8a, after high nitrogen treatment, *CsaV4_3G000307* was significantly upregulated at 2 and 4 h in the high NUE line, whereas no significant up-regulation was observed in the low NUE line, suggesting that *CsaV4_3G000307* may play a more active role in the initial high nitrogen response in the high NUE line. A remarkable down regulation was observed in both lines at time point 8 h. The expression level recovered again at time points 12 h and 24 h in both the high NUE and low NUE lines. At the time point 30 h, the expression level declined remarkably in the low NUE line but maintained normal expression in the high NUE line.

As illustrated in Figure 8c, *CsaV4_1G002492* showed no differences in expression before high nitrogen treatment. A significant down-regulation in the high NUE line was observed at all time points except at 8 h, and a significant up-regulation in the low NUE line was observed at all time points. Figure 8e revealed that *CsaV4_2G003460* exhibited a higher expression in the low NUE line at 0, 2, 8, and 30 h than that in high NUE line. According to Figure 8g, *CsaV4_7G001709* showed up-regulation at 2 h and 4 h, but down-regulation at other time points in the high NUE line, while in low NUE line, it was up-regulated at 4 h, but down-regulation at other time points.

This analysis demonstrates the distinct expression profiles of *CsaV4_3G000307*, *CsaV4_7G001709*, *CsaV4_2G003460*, and *CsaV4_1G002492* in the high NUE and low NUE lines. *CsaV4_3G000307* and *CsaV4_7G001709* were suggested to be related to enhancing NUE, whereas *CsaV4_2G003460* and *CsaV4_1G002492* appeared to be reducing NUE. Linear regression analysis confirmed a high consistency between qPCR results and transcriptomic data (Figure 8b,d,f,h), verifying the reliability of the transcriptomic data.

### 3.6. Protein Interaction Prediction for CsaV4_3G000307

**Figure 9:**
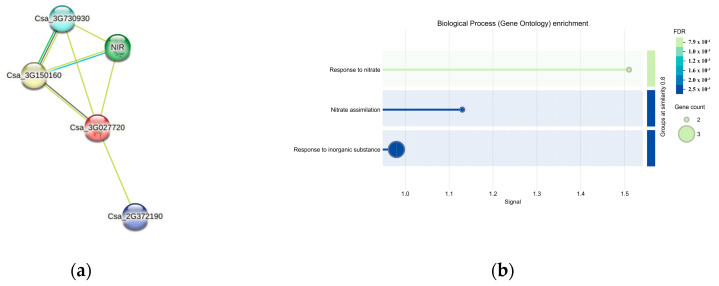
(**a**) Protein interaction network for the gene *CsaV4_3G000307*. Nodes represent proteins, each encapsulating all proteins from a single gene locus, including splice isoforms and post-translational modifications. Edges denote specific and meaningful protein–protein interactions, highlighting the precision of the depicted associations. (**b**) Heatmap of Gene Enrichment Analysis. This illustrates False Discovery Rate (FDR) values across various categories, with a color gradient from lighter green (lower FDR values) to darker blue (higher values) indicating the significance of gene enrichment. Circles on the heatmap represent gene counts in each category, varying sizes correspond to gene numbers: smaller circles for two genes, larger circles for three genes.

Four interacting proteins were identified through protein interaction predictions for *CsaV4_3G000307* (*Csa_3G027720*) *(*Figure 9a). Notably, *NIR* and the protein of *Csa_2G372190* were recognized within the nitrogen metabolism pathway, as indicated in the KEGG database (Figure 9b). The protein of *Csa_3G730930* was linked to the ammonium transmembrane transporter complex. The protein of *Csa_2G372190* was characterized as a non-specific serine/threonine protein kinase within the protein kinase superfamily. The protein of *Csa_3G150160* was characterized as glutamine synthetase, which plays a crucial role in nitrogen assimilation.

### 3.7. Protein Interaction Prediction for CsaV4_1G002492

**Figure 10:**
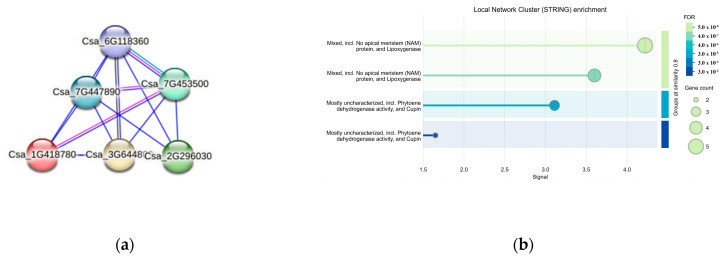
(**a**) Protein structural and interaction network for the gene *CsaV4_1G002492*. Nodes are synonymous with proteins. (**b**) Heatmap of Gene Enrichment Analysis.

Five interacting proteins were identified through protein interaction predictions for *CsaV4*_1G002492 (Csa_*1G418780*) (Figure 10a). *Csa_3G644800* contained the Cupin 1 domain. The proteins of *Csa_2G296030* and *Csa_7G447890* are characterized as having undefined functions. The protein of *Csa_6G118360* possessed a C2H2-type zinc finger domain, which is typically involved in the transcriptional regulation of plants. The protein of *Csa_7G453500* contained a plastocyanin-like domain, which is related to photosynthesis. Figure 10b highlighted the enriched functions of these proteins, including No apical meristem (NAM) protein, Lipoxygenase, Phytoene dehydrogenase activity, and the Cupin domain.

### 3.8. Protein Interaction Prediction of CsaV4_2G003460

**Figure 11:**
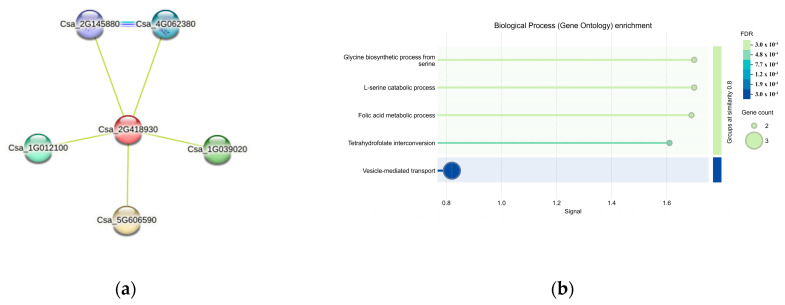
Protein interaction network for the gene *CsaV4_2G003460*. (**a**) Nodes are synonymous with proteins; (**b**) Heatmap of Gene Enrichment Analysis.

Five interacting proteins were identified through protein interaction predictions for *CsaV4_2G003460* (*Csa_2G418930*) *(*Figure 11a). Among them, the proteins of *Csa_5G606590*, *Csa_1G039020*, and *Csa_1G012100* contained the t-SNARE coiled-coil homology domain, typically crucial in vesicular trafficking processes like endocytosis and exocytosis, essential for cellular transport mechanisms. The proteins of *Csa_4G062380* and *Csa_2G145880* were identified as serine hydroxymethyltransferases, directly involved in amino acid metabolism. As illustrated in Figure 11b, the enriched gene functions primarily included the glycine biosynthetic process from serine, L-serine catabolic process, folic acid metabolic process, tetrahydrofolate interconversion, and vesicle-mediated transport.

### 3.9. Protein Interaction Prediction of CsaV4_7G001709

**Figure 12:**
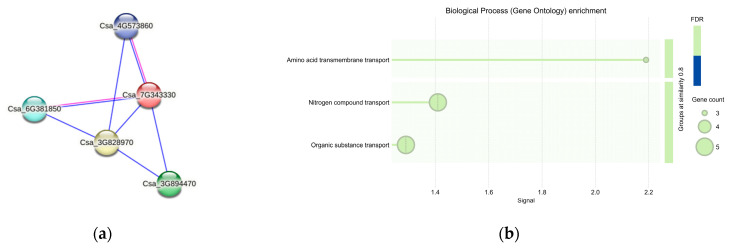
(**a**) Protein interaction network for the gene *CsaV4_7G001709*. Nodes are synonymous with proteins. (**b**) Heatmap of Gene Enrichment Analysis.

Four interacting proteins were identified through protein interaction predictions for *CsaV4_7G001709* (*Csa_7G343330*) (Figure 12a). Among these, the protein of the gene *Csa_3G828970* has undefined characteristics. The proteins of *Csa_3G894470*, *Csa_6G381850*, and *Csa_4G573860* all contained the Aa_trans (amino acid transporter) domain, which typically plays a crucial role in amino acid transport processes vital for nitrogen assimilation and metabolism. As shown in Figure 12b, the functions enriched for these proteins included amino acid transmembrane transport, nitrogen compound transport, and organic substance transport, which are all closely related to nitrogen metabolism.

### 3.10. Transcriptomic SNP and INDEL Analysis of the Key Genes

**Figure 13:**
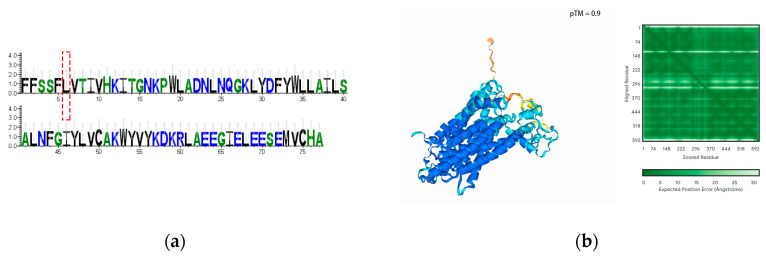
Protein sequence and structural analysis in the high NUE line. (**a**) The protein sequence logo, where the conservation of amino acids at specific positions within the sequence is represented by the height of letters. The red box marks the start position of the frame-shift mutation. (**b**) The three-dimensional model of the protein structure. The accompanying green matrix represents the predicted interaction strength or structural stability between amino acid residues within the protein, with darker green indicating higher prediction accuracy or stability, and lighter green indicating lower accuracy or stability.

**Figure 14:**
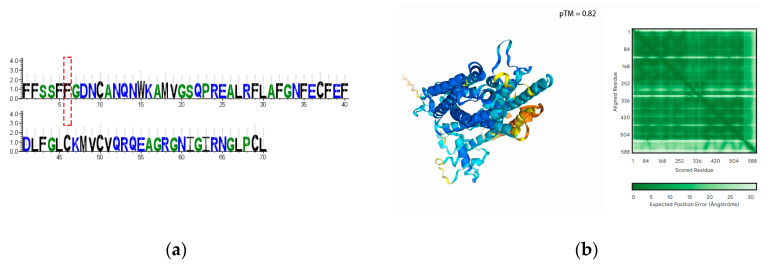
Protein sequence and structural analysis in the low NUE line. (**a**) The protein sequence logo. The red box marks the start position of the frame-shift mutation. (**b**) The three-dimensional model of the protein structure.

The structural integrity of the four genes was analyzed at the transcriptional level for the presence of SNPs and INDELs. No SNPs or INDELs were found in *CsaV4_2G003460*, *CsaV4_1G002492* and *CsaV4_7G001709* in either the high NUE or low NUE lines. However, an INDEL was identified in *CsaV4_3G000307,* causing a frame-shift mutation that occurs at the 522nd leucine exclusively in the low NUE line (Figure 13a and Figure 14a). Structural analysis for both the high NUE and low NUE lines revealed that the frame-shift mutation impacted the compactness of the protein, potentially altering its functionality (Figure 13b and Figure 14b).

## 4. Discussion

Nitrogen is essential for synthesizing chlorophyll, amino acids, and nucleic acids, making it indispensable for plant growth and development [61,62,63]. However, a significant amount of nitrate nitrogen is lost from the soil due to plant uptake and leaching by irrigation or rain, reducing nitrogen use efficiency [64]. By improving plant NUE, we can significantly reduce cultivation costs and mitigate nitrate pollution.

In this study, root transcriptomic responses to high nitrogen were evaluated across multiple time points in two contrasting cucumber genotypes, high NUE and low NUE, selected based on extensive field and laboratory research. A multitude of DEGs were identified at seven time points within both lines, affirming the validity of the material selection and laying a solid foundation for subsequent analyses. To isolate genes associated with NUE, WGCNA was employed to categorize 15,180 genes into 35 modules, ranging from the smallest gray module containing 48 genes to the largest turquoise module encompassing over 4000 genes. This modular division reflected the complexity of gene expression in the high NUE and low NUE lines. Notably, five modules showed expression patterns significantly exclusive to either the high NUE or low NUE lines. The purple module exhibited the highest correlation coefficient at 0.99, followed by skyblue at 0.95, darkorange at 0.71, cyan at 0.72, and lightcyan at 0.68. Among these, the purple module was particularly notable with 241 genes, 83.4% of which were differentially expressed between the two lines, whereas the cyan module, comprising 200 genes, showed only 53.5% differential expression, reducing its significance. Despite the skyblue module being highly correlated with the high NUE line, it contained the fewest genes, 86, with 63.95% showing differential expression, which suggests that a few key genes play a critical role in enhancing this module’s relevance to NUE. Indeed, gene *CsaV4_7G001709* from the skyblue module was annotated as UPS2, which is a protein rich in nitrogen and directly related to NUE [57]. Additionally, genes *CsaV4_2G003460* and *CsaV4_1G002492* from the lightblue module are annotated as glutamate receptor 3.5 and 2.2 and are directly involved in nitrogen metabolism [43,44]. *CsaV4_3G000307* and *CsaV4_7G001709* may play positive regulatory roles in the NUE process. In contrast, *CsaV4_2G003460* and *CsaV4_1G002492* typically showed higher expression levels in the low NUE line, indicating their potential negative regulatory effects on NUE. Additional analysis explored whether these expression differences stemmed from structural changes within the genes, such as SNPs or INDELs, since these genetic variations are acknowledged as potential factors in phenotypic segregation [47].

Transcriptome analysis revealed a frame-shifting INDEL mutation exclusively in *CsaV4_3G000307* within the low NUE line; no missense mutation SNPs were detected in any of other the three genes across either line. Both missense mutation SNPs and frame-shifting INDELs can modify amino acid sequences, potentially affecting protein functionality [48], thus structural predictions were conducted for *CsaV4_3G000307*’s sequences before and after the mutation. Post-mutation, the protein structure appeared looser, which might influence protein binding and functionality. However, whether this INDEL actually causes functional changes requires further verification, such as whether the gene also exhibits the same INDEL in the genome, whether the presence of this INDEL in the transcriptome genuinely affects protein coherence, and whether the occurrence of this INDEL is due to alternative splicing or sequencing errors; these aspects all necessitate subsequent verification.

The homolog gene of *CsaV4_3G000307* in *Arabidopsis thaliana*, *AtNPF6.3/NRT1.1*, plays a pivotal role in detecting external nitrogen levels and transporting extracellular nitrogen into cells [49]. Positioned on the plasma membrane, *AtNPF6.3* enables nitrate transport from the extracellular environment into the cytoplasm by facilitating phosphorylation, which activates nitrate ion channels [50]. This function is conserved across species, as demonstrated by the nitrate transport activity of *AtNPF6.3* homologs in rice and maize [15,51]. Consequently, *CsNPF6.3/NRT1.1* is likely to be integral to the nitrate transport process in cucumber as well. Furthermore, *AtNPF6.3* is implicated in various other functions, including root development regulation via auxin transport, nitrate signal transduction, nutrient signal integration, and plant–microbe interaction regulation, highlighting its essential role in plant stress resistance and developmental processes [52,53,54]. *NRT1.1* has been implicated in several regulatory networks; for example, *STOP1* activation enhances *NRT1.1*-mediated nitrate absorption [55], while *NRT1.1* collaborates with *NLP7* in nitrogen sensing and transport [56]. It also partners with *NRT2.1* to facilitate nitrate transport [65], and is negatively regulated by abscisic acid signaling, which phosphorylates *NRT1.1* via SnRK2s, inhibiting nitrate uptake [66]. Interaction predictions for *CsaV4_3G000307* identified four potential interacting genes or proteins: *Csa_2G372190*, *Csa_3G730930*, and *Csa_3G150160* are identified as NIRs, where *Csa_3G150160* serves as glutamine synthetase (GS). Once nitrate is transported to relevant sites, enzymes such as nitrate reductase (NR), NIR, GS, and glutamate synthase (GOGAT) transform it into amino acids that fulfill diverse biological functions [67]. *Csa_3G730930* acts as an AMT, which is essential for the nitrogen needs of plants, particularly in flood-prone or acidic soils where ammonium is prevalent [68]. The protein of *Csa_2G372190*, which is identified as a non-specific serine/threonine protein kinase, is part of the protein kinase superfamily [69,70,71]. The protein kinase translated by *Csa_2G372190* may stimulate the expression of certain pathways. If there are also differences in the expression of this gene between high NUE and low NUE lines, it could be related to the differential expression of *CsaV4_3G000307* in the two lines. Additionally, *Csa_3G730930* and *CsaV4_3G000307* may have a synergistic effect on nitrogen absorption. The variable expression of *CsaV4_3G000307* in the high NUE and low NUE lines might lead to changes in nitrogen absorption and transport efficiency, thereby impacting the role of proteins such as GS and NIR in the nitrogen assimilation process.

The proteins of *CsaV4_1G002492* and *CsaV4_2G003460* belong to GLRs. In Arabidopsis, overexpression of *AtGLR2* leads to symptoms of Ca^2^+ deficiency [45], and Ca^2^+ is closely related to the absorption and transport of nitrogen within cells [56]. *AtGLR1.1* is a regulator of carbon and nitrogen metabolism in *Arabidopsis*, controlling seed germination by influencing abscisic acid (ABA) to regulate carbon and nitrogen metabolism [46]. The enrichment of *CsaV4_1G002492*’s predicted interaction proteins, such as NAM protein, lipoxygenase, and phytoene dehydrogenase are directly or indirectly related to nitrogen metabolism [72,73,74]. Among them, the protein of *Csa_6G118360* possessed a C2H2-type zinc finger domain, which typically involved in the transcriptional regulation of plants and the regulation of *Csa_6G118360* may affect the differential expression of the *CsaV4_1G002492* gene between the two extreme lines. The enriched *interaction* gene functions of *CsaV4_2G003460* include the glycine biosynthetic process from serine, L-serine catabolic process, folic acid metabolic process, tetrahydrofolate interconversion, and vesicle-mediated transport, which are also directly or indirectly related to nitrogen metabolism [75,76,77]. The proteins of *CsaV4_1G002492* and *CsaV4_2G003460* may have a synergistic effect in the process of nitrogen metabolism.

The protein of *CsaV4_7G001709* is UPS2. In soybean, the UPS1 protein plays a crucial role in managing the allocation of ureides from the source to the sink tissues. Enhancing the expression of UPS1 significantly increases the nitrogen supply to the sink tissues of soybean, which in turn contributes to higher grain yields [58,59,75]. In wheat, TaUPS1 and TaUPS2.1 are involved in nitrogen distribution and NUE. Three of the predicted interaction proteins (the proteins of *Csa_3G894470*, *Csa_6G381850* and *Csa_4G573860*) contained the Aa_trans (amino acid transporter) domain and the functions enriched for these proteins include amino acid transmembrane transport, nitrogen compound transport, and organic substance transport, participating in nitrogen metabolism [78,79,80]. Overall, the enrichment of these functions indicates that the associated genes play a direct role in regulating plant nitrogen nutrition and metabolism, enhancing the four key genes potential significance in NUE.

Future research will employ transgenic technologies such as gene silencing, knockout, and over-expression to elucidate the roles of the key genes that regulate NUE in cucumbers. The investigation will extend to mining upstream and downstream genes and performing network analysis, enhancing the understanding of the genetic networks of NUE in cucumber.

## 5. Conclusions

The study analyzed transcriptomic data through WGCNA combined with other methods, identifying four key genes that are potentially instrumental in enhancing NUE. This investigation offers potential loci for molecular breeding and lays the groundwork for ensuing genetic and molecular inquiries dedicated to enhancing NUE in cucumbers.

## Figures and Tables

**Table 1:** Gene information and functional annotation of the 25 selected genes.

Gene ID	Gene Description	Organism Species	Module	Related References
*CsaV4_1G000167*	BALDH—Benzaldehyde dehydrogenase, mitochondrial	*Antirrhinum majus*	lightcyan	
*CsaV4_1G002491*	TAL2—Transaldolase 2	*Streptomyces coelicolor*	darkorange	
*CsaV4_1G002492*	GLR22—Glutamate receptor 2.2	*Arabidopsis thaliana*	lightcyan	[43,44,45,46]
*CsaV4_1G002522*	NAS4—Probable nicotianamine synthase 4	*Arabidopsis thaliana*	lightcyan	
*CsaV4_1G003819*	TENAC—Bifunctional TH2 protein, mitochondrial	*Arabidopsis thaliana*	purple	
*CsaV4_2G000093*	HXK2—Hexokinase-2	*Arabidopsis thaliana*	purple	
*CsaV4_2G000502*	NAS3—Nicotianamine synthase 3	*Arabidopsis thaliana*	lightcyan	
*CsaV4_2G000612*	MDL3— (R)-mandelonitrile lyase 3	*Prunus serotina*	darkorange	
*CsaV4_2G003460*	GLR35—Glutamate receptor 3.5	*Arabidopsis thaliana*	lightcyan	[43,44,45,46]
*CsaV4_2G003519*	AB14B—ABC transporter B family member 14	*Arabidopsis thaliana*	lightcyan	
*CsaV4_3G000307*	NPF6.3—Protein NRT1/PTR FAMILY 6.3	*Arabidopsis thaliana*	darkorange	[47,48,49,50,51,52,53,54,55,56]
*CsaV4_3G001073*	AB9C—ABC transporter C family member 9	*Arabidopsis thaliana*	lightcyan	
*CsaV4_3G003070*	SCP35—Serine carboxypeptidase-like 35	*Arabidopsis thaliana*	purple	
*CsaV4_3G004703*	SOX—Probable sarcosine oxidase	*Arabidopsis thaliana*	purple	
*CsaV4_3G004765*	ISOA3—Isoamylase 3, chloroplastic	*Arabidopsis thaliana*	purple	
*CsaV4_4G000627*	ISPF—2-C-methyl-D-erythritol 2,4-cyclodiphosphate synthase, chloroplastic	*Arabidopsis thaliana*	purple	
*CsaV4_4G000995*	RIBA1—Bifunctional riboflavin biosynthesis protein, chloroplastic	*Arabidopsis thaliana*	lightcyan	
*CsaV4_4G002807*	ZIP3—Zinc transporter 3	*Arabidopsis thaliana*	lightcyan	
*CsaV4_5G000080*	CHIA—Acidic endochitinase	*Cucumis sativus*	purple	
*CsaV4_6G000223*	PER52—Peroxidase 52	*Arabidopsis thaliana*	darkorange	
*CsaV4_6G000691*	RBL13—RHOMBOID-like protein 13	*Arabidopsis thaliana*	purple	
*CsaV4_6G002589*	ACCO1—1-aminocyclopropane-1-carboxylate oxidase 1	*Cucumis melo*	lightcyan	
*CsaV4_6G002776*	PIP21—Probable aquaporin PIP2-1	*Oryza sativa* subsp. *japonica*	darkorange	
*CsaV4_7G000732*	XYN4—Endo-1,4-beta-xylanase 4	*Arabidopsis thaliana*	purple	
*CsaV4_7G001709*	UPS2—Ureide permease 2	*Arabidopsis thaliana*	skyblue	[57,58,59,60]

## Data Availability

The raw Illumina sequence reads have been deposited into the National Center for Biotechnology Information under sequence read archive (SRA) PRJNA1175851.

## Supplementary Materials

The following supporting information can be downloaded at: https://www.mdpi.com/article/10.3390/genes15121505/s1, Table S1: The Hoagland nutrient solution; Table S2: The primer sequence information; Table S3: GO Result of Purple Module; Table S4: KEGG Result of Purple Module; Table S5: GO Result of Lightcyan Module; Table S6: KEGG Result of Lightcyan Module; Table S7: GO Result of Darkorange Module; Table S8: KEGG Result of Darkorange Module; Table S9: GO Result of Cyan Module; Table S10: KEGG Result of Cyan Module; Table S11: GO Result of Skyblue Module; Table S12: KEGG Result of Skyblue Module; Figure S1: Gene co-expression modules identified through WGCNA; Figure S2: Venn Diagram of DEGs in Purple Module Across Time Points; Figure S3: GO enrichment analysis of DEGs in Purple Module; Figure S4: KEGG Pathway enrichment analysis of DEGs in Purple Module; Figure S5: Venn Diagram of DEGs in Lightcyan Module; Figure S6: GO enrichment analysis of DEGs in Lightcyan Module; Figure S7: KEGG enrichment analysis of DEGs in Lightcyan Module; Figure S8: Venn Diagram of DEGs in Darkorange Module; Figure S9: GO enrichment analysis of DEGs in Darkorange Module; Figure S10: KEGG enrichment analysis of DEGs in Darkorange Module; Figure S11: Venn Diagram of DEGs in Cyan Module; Figure S12: GO enrichment analysis of DEGs in Cyan Module; Figure S13: KEGG enrichment analysis of DEGs in Cyan Module; Figure S14: Venn Diagram of DEGs in Skyblue Module; Figure S15: GO enrichment analysis of DEGs in Skyblue Module; Figure S16: KEGG enrichment analysis of DEGs in Skyblue Module; File S1: The protein sequence of the key genes.
