# Supplementary material for: Screening Key Genes Related to Nitrogen Use Efficiency in Cucumber Through Weighted Gene Co-Expression Network Analysis
Source: Genes (Basel). 2024 Nov 24;15(12):1505. doi: 10.3390/genes15121505 (PMC11675882; doi:10.3390/genes15121505)
Supplement: Supplementary file 1 [file genes-15-01505-s001.zip › Supplemental Figures.pdf]

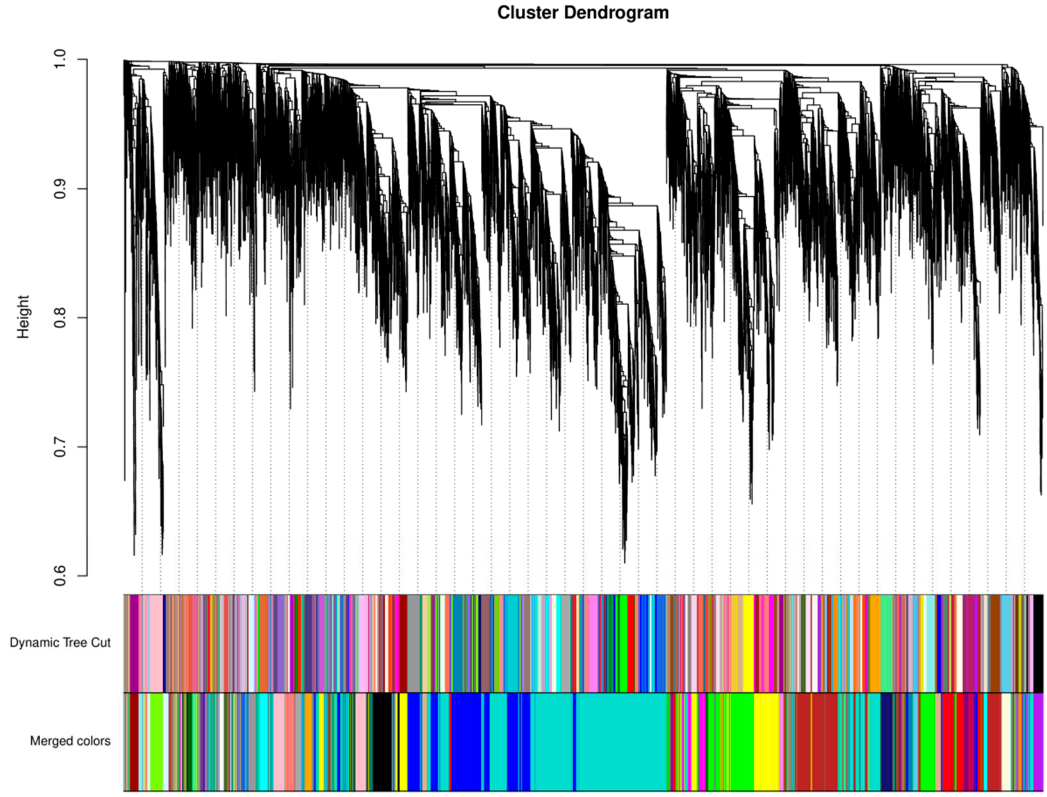

Figure S1. Gene co-expression modules identified through WGCNA. Each module represented by a unique color, grouping genes with similar expression patterns. Top: Gene clustering tree, each leaf represents a gene, modules appear as branches. Middle: Dynamic Tree Cut for module identification, colors denote distinct modules. Bottom: 'Merged colors' show merged modules with a dissimilarity coefficient below 0.25.

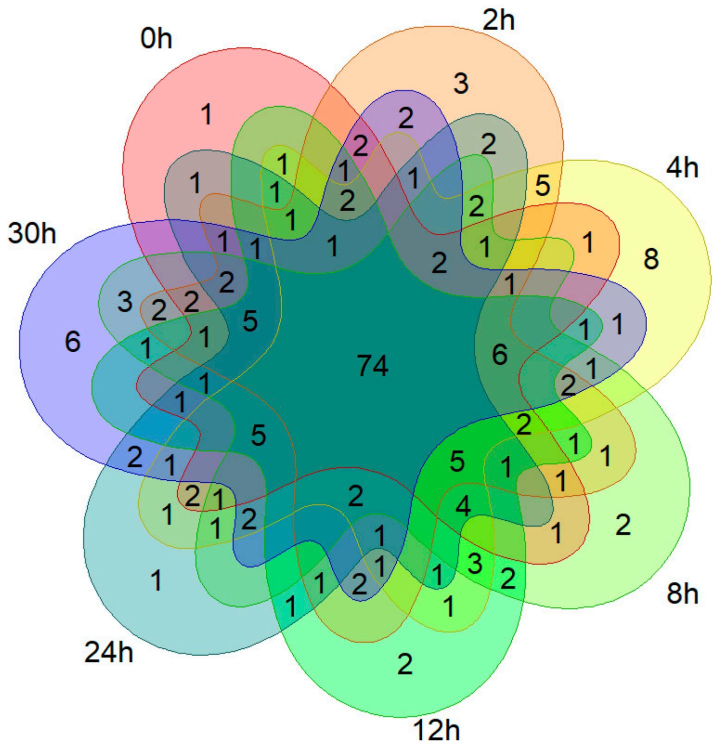

Figure S2. Venn Diagram of DEGs in Purple Module Across Time Points. Venn diagram depicting the overlapping and unique DEGs at different time points (0h, 2h, 4h, 8h, 12h, 24h, and 30h). The central number indicates genes common to all time points, while the surrounding numbers represent genes that are specific to each time point.

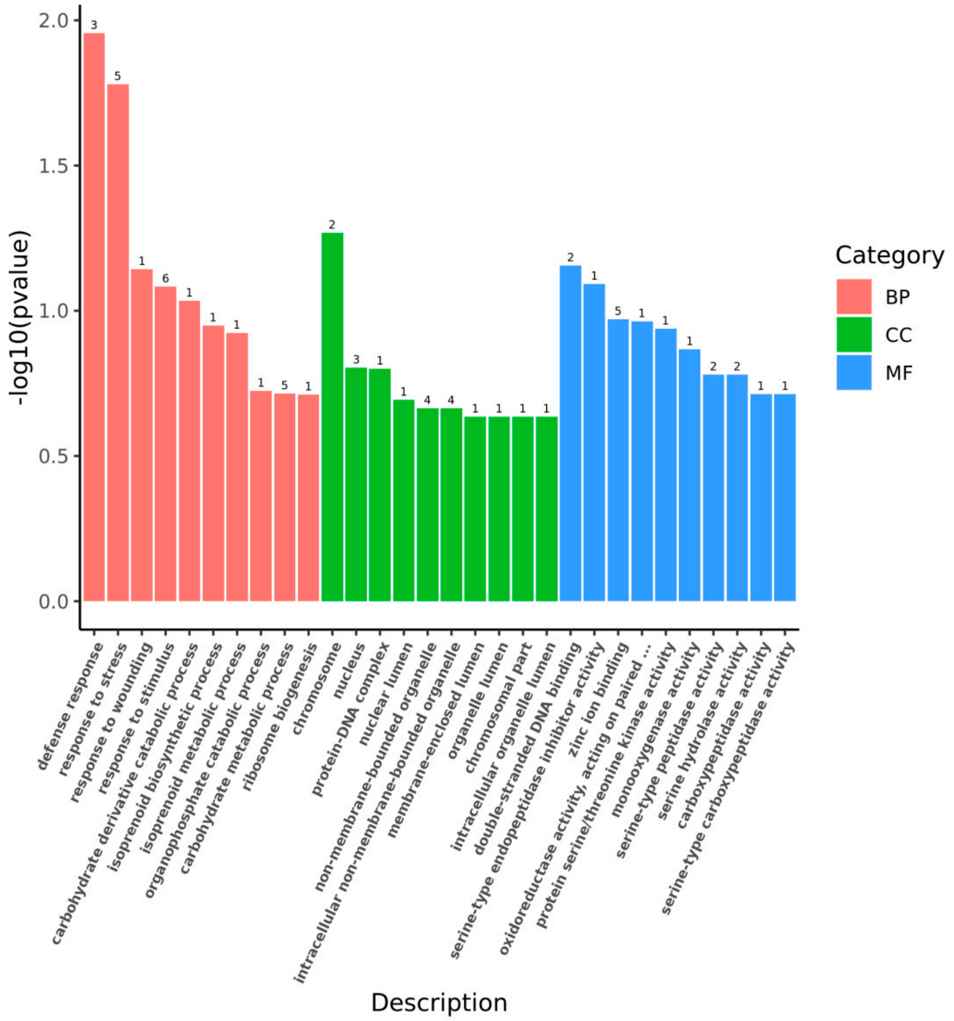

Figure S3. GO enrichment analysis of DEGs in Purple Module. Categorizes enriched biological processes (BP), cellular components (CC), and molecular functions (MF) based on their significance (-$\log_{10}$ p-value).

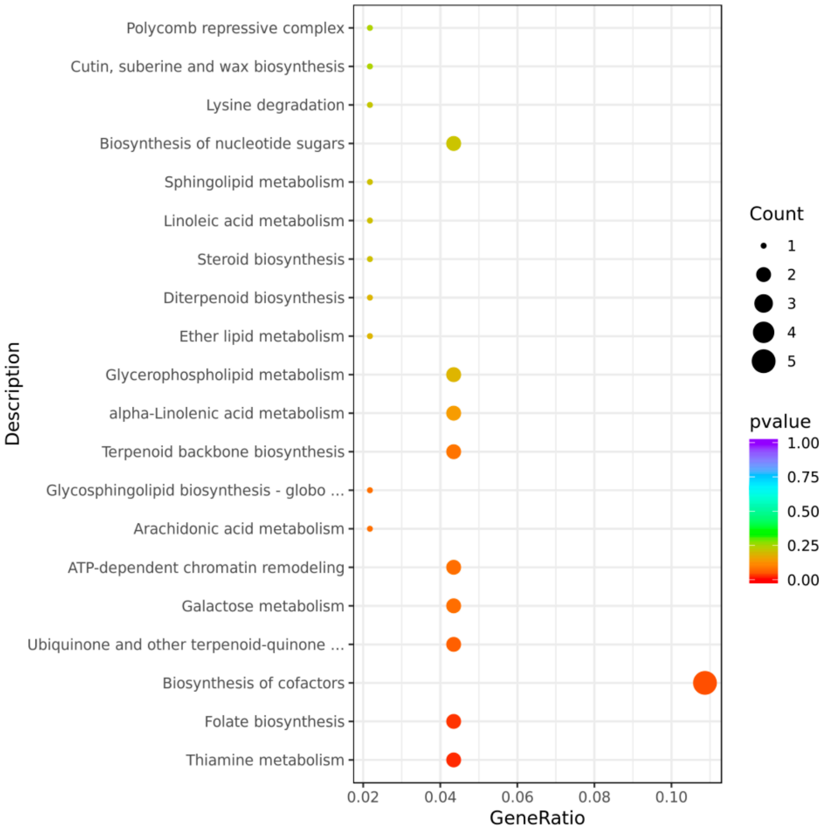

Figure S4. KEGG Pathway enrichment analysis of DEGs in Purple Module. The size of the dots reflects the count of genes in each pathway, while the color indicates the p-value, with red showing higher significance.

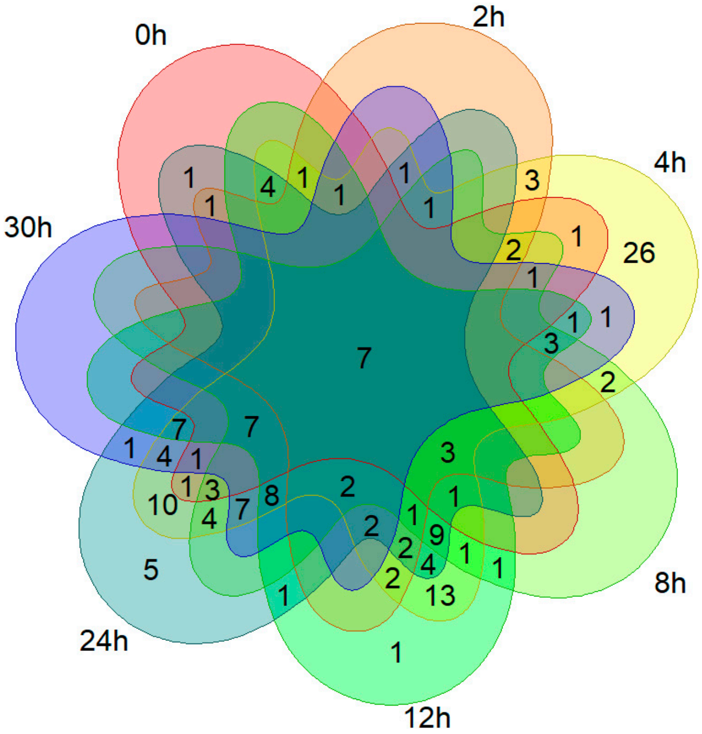

Figure S5. Venn Diagram of DEGs in Lightcyan Module.

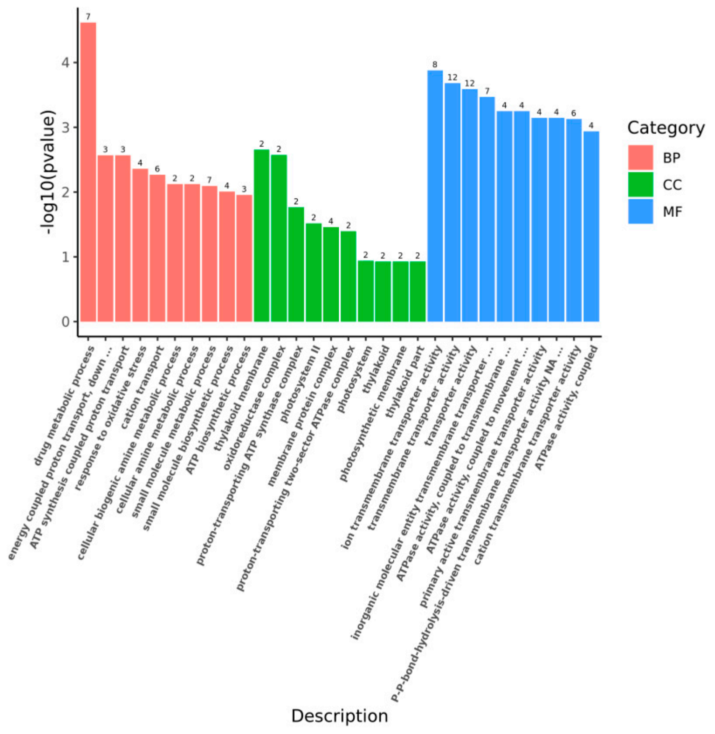

Figure S6. GO enrichment analysis of DEGs in Lightcyan Module.

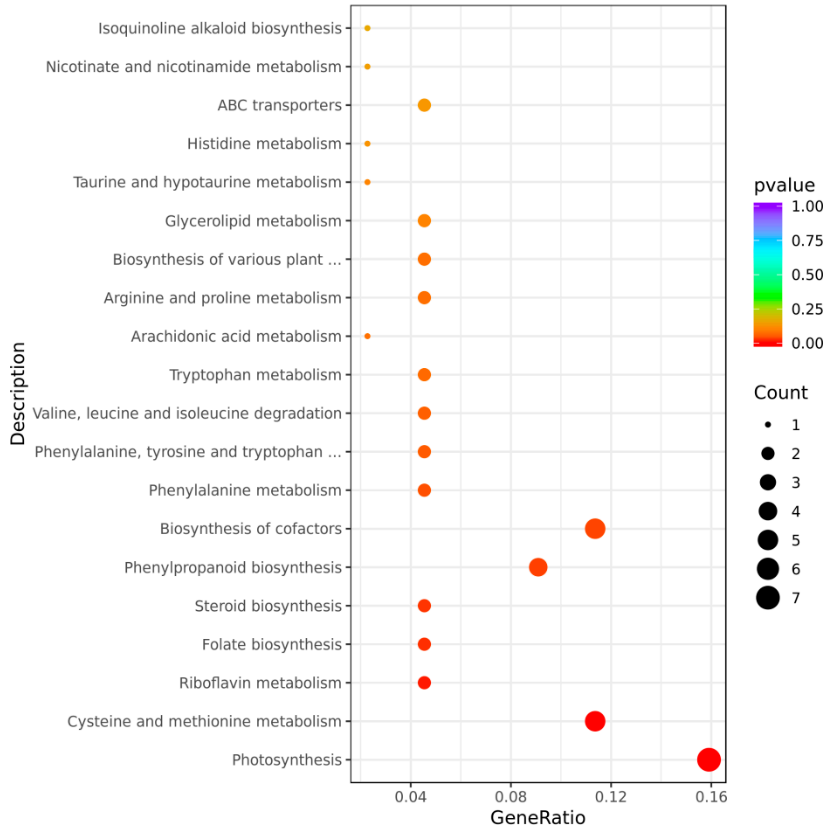

Figure S7. KEGG enrichment analysis of DEGs in Lightcyan Module.

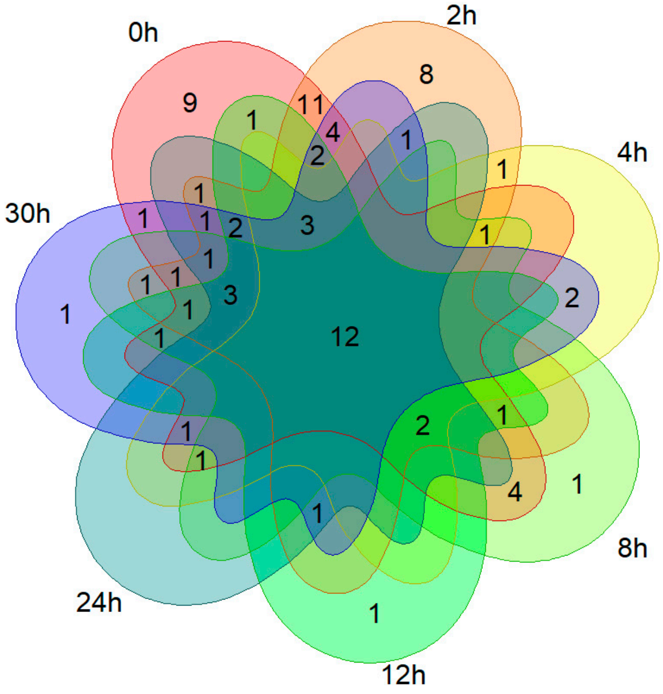

Figure S8. Venn Diagram of DEGs in Darkorange Module.

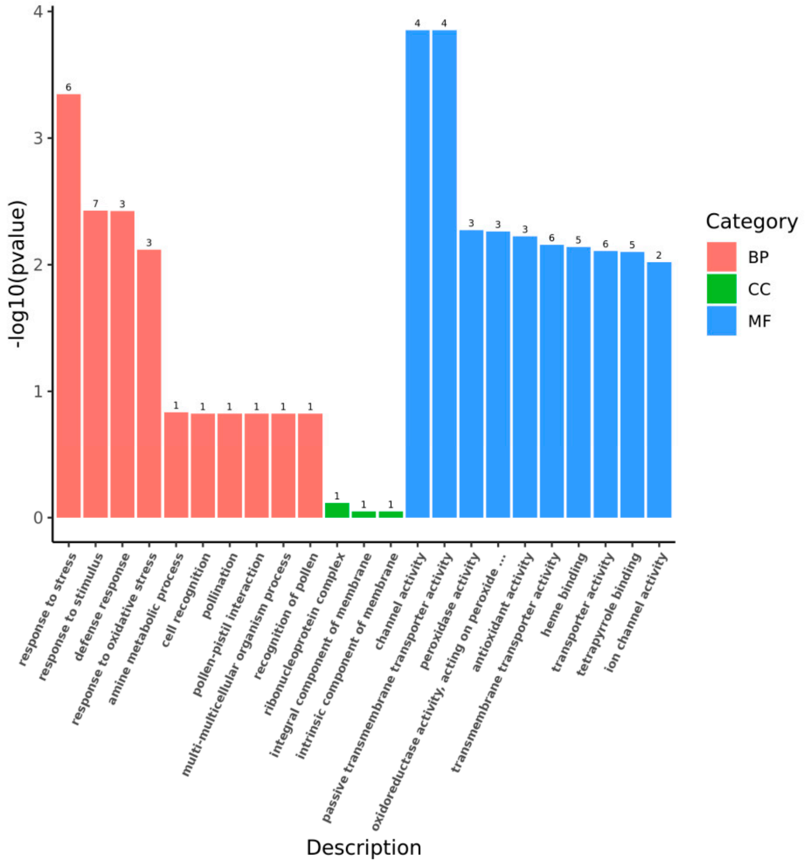

Figure S9. GO enrichment analysis of DEGs in Darkorange Module.

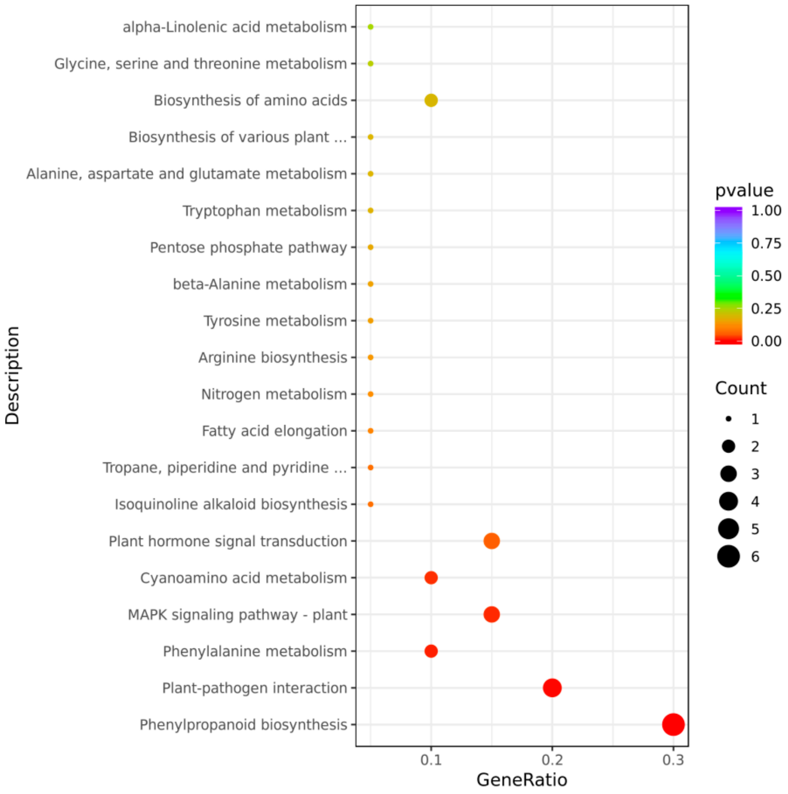

Figure S10. KEGG enrichment analysis of DEGs in Darkorange Module.

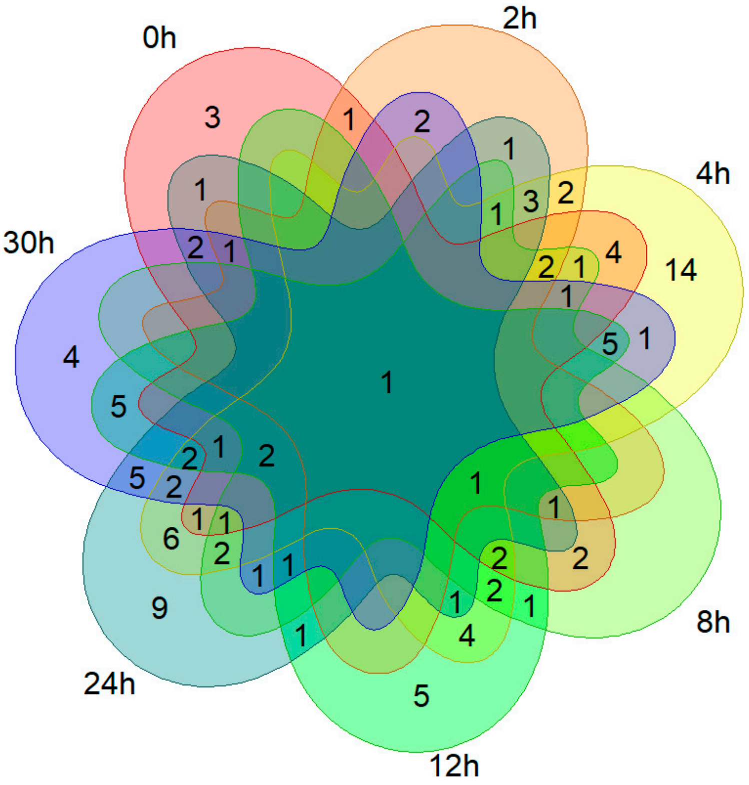

Figure S11. Venn Diagram of DEGs in Cyan Module.

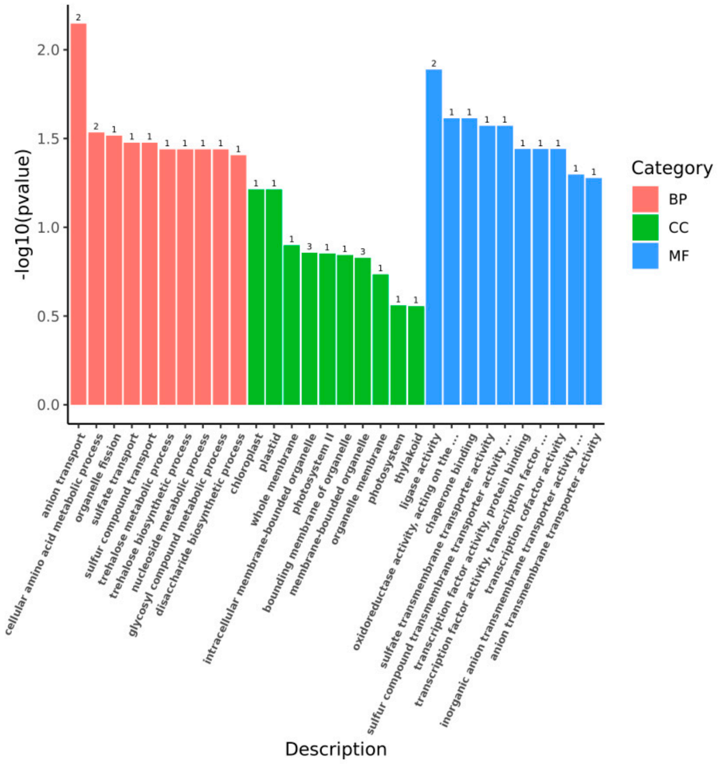

Figure S12. GO enrichment analysis of DEGs in Cyan Module.

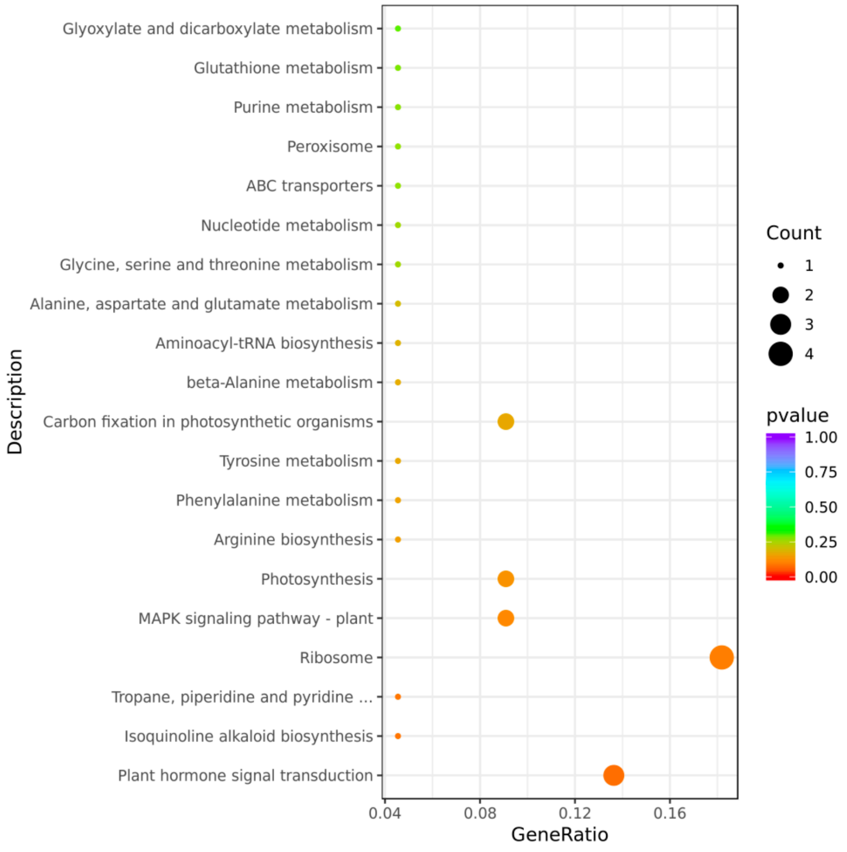

Figure S13. KEGG enrichment analysis of DEGs in Cyan Module.

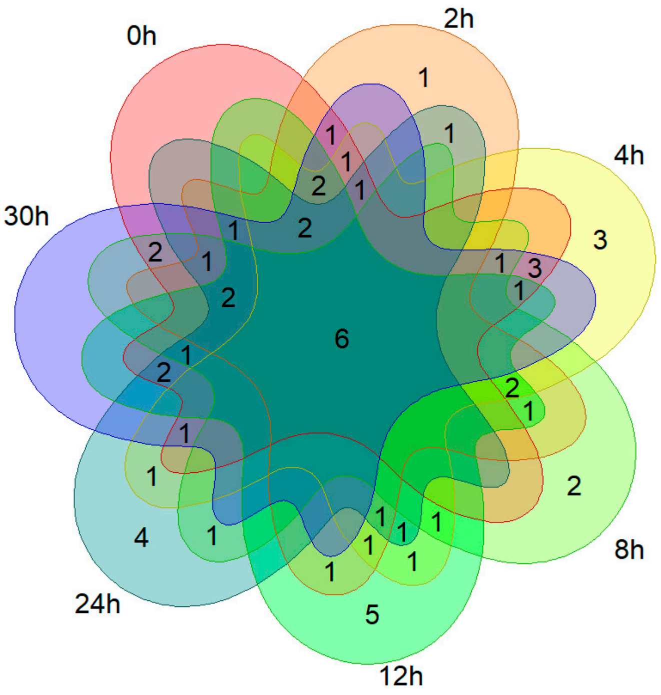

Figure S14. Venn Diagram of DEGs in Skyblue Module.

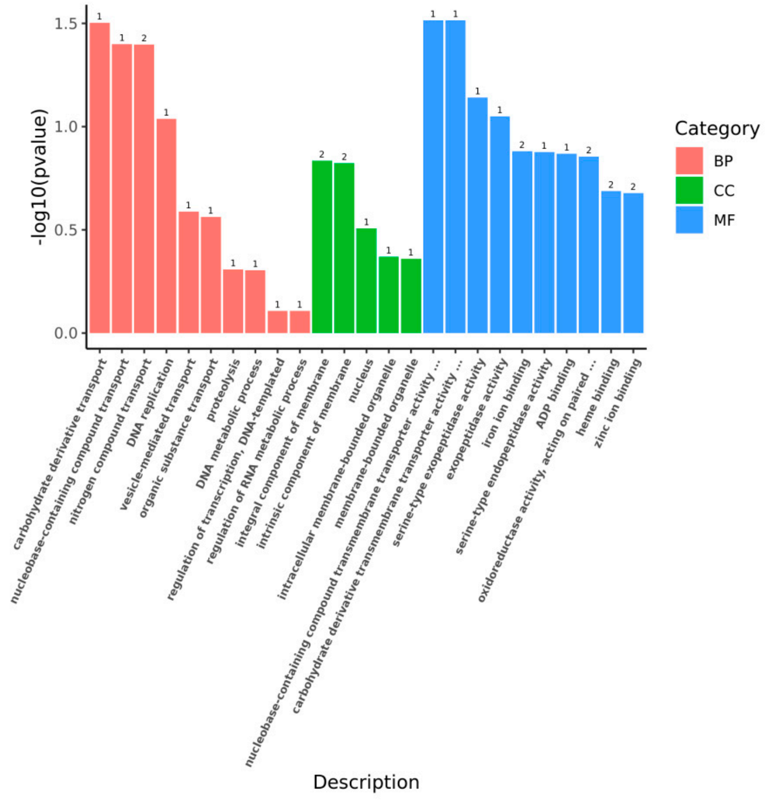

Figure S15. GO enrichment analysis of DEGs in Skyblue Module.

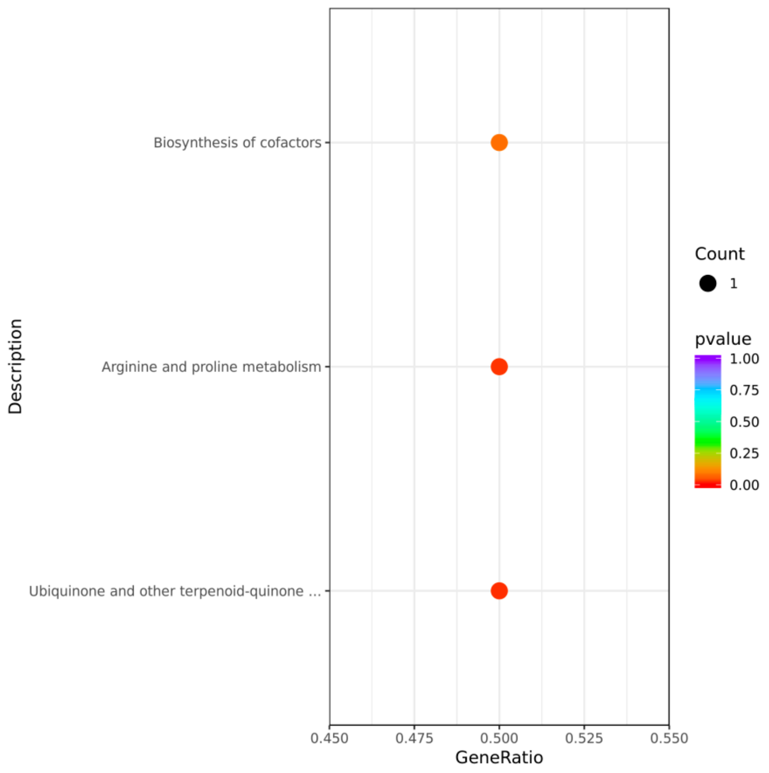

Figure S16. KEGG enrichment analysis of DEGs in Skyblue Module.
